# Diagnostic Performance of a Deep Learning Model Deployed at a National COVID-19 Screening Facility for Detection of Pneumonia on Frontal Chest Radiographs

**DOI:** 10.3390/healthcare10010175

**Published:** 2022-01-17

**Authors:** Jordan Z. T. Sim, Yong-Han Ting, Yuan Tang, Yangqin Feng, Xiaofeng Lei, Xiaohong Wang, Wen-Xiang Chen, Su Huang, Sum-Thai Wong, Zhongkang Lu, Yingnan Cui, Soo-Kng Teo, Xin-Xing Xu, Wei-Min Huang, Cher-Heng Tan

**Affiliations:** 1 Department of Diagnostic Radiology, Tan Tock Seng Hospital, 11, Jalan Tan Tock Seng, Singapore 308433, Singapore; Yonghan_ting@ttsh.com.sg (Y.-H.T.); chen_wen_xiang@ttsh.com.sg (W.-X.C.); cher_heng_tan@ttsh.com.sg (C.-H.T.); 2 Healthcare-MedTech Division & Visual Intelligence Department, Institute for Infocomm Research, A*STAR, 1 Fusionopolis Way, Singapore 138632, Singapore; ytang016@e.ntu.edu.sg (Y.T.); wang_xiaohong@i2r.a-star.edu.sg (X.W.); huangs@i2r.a-star.edu.sg (S.H.); zklu@i2r.a-star.edu.sg (Z.L.); wmhuang@i2r.a-star.edu.sg (W.-M.H.); 3 Institute of High Performance Computing, A*STAR, 1 Fusionopolis Way, Singapore 138632, Singapore; Feng_Yangqin@ihpc.a-star.edu.sg (Y.F.); Lei_Xiaofeng@ihpc.a-star.edu.sg (X.L.); wsumthai@gmail.com (S.-T.W.); cuiyn@ihpc.a-star.edu.sg (Y.C.); sookng@gmail.com (S.-K.T.); xuxinx@ihpc.a-star.edu.sg (X.-X.X.); 4 Lee Kong Chian School of Medicine, Nanyang Technological University, 11, Mandalay Road, Singapore 308232, Singapore

**Keywords:** COVID-19, pneumonia, X-ray, mass chest, intelligence, artificial, learning, deep

## Abstract

(1) Background: Chest radiographs are the mainstay of initial radiological investigation in this COVID-19 pandemic. A reliable and readily deployable artificial intelligence (AI) algorithm that detects pneumonia in COVID-19 suspects can be useful for screening or triage in a hospital setting. This study has a few objectives: first, to develop a model that accurately detects pneumonia in COVID-19 suspects; second, to assess its performance in a real-world clinical setting; and third, by integrating the model with the daily clinical workflow, to measure its impact on report turn-around time. (2) Methods: The model was developed from the NIH Chest-14 open-source dataset and fine-tuned using an internal dataset comprising more than 4000 CXRs acquired in our institution. Input from two senior radiologists provided the reference standard. The model was integrated into daily clinical workflow, prioritising abnormal CXRs for expedited reporting. Area under the receiver operating characteristic curve (AUC), F1 score, sensitivity, and specificity were calculated to characterise diagnostic performance. The average time taken by radiologists in reporting the CXRs was compared against the mean baseline time taken prior to implementation of the AI model. (3) Results: 9431 unique CXRs were included in the datasets, of which 1232 were ground truth-labelled positive for pneumonia. On the “live” dataset, the model achieved an AUC of 0.95 (95% confidence interval (CI): 0.92, 0.96) corresponding to a specificity of 97% (95% CI: 0.97, 0.98) and sensitivity of 79% (95% CI: 0.72, 0.84). No statistically significant degradation of diagnostic performance was encountered during clinical deployment, and report turn-around time was reduced by 22%. (4) Conclusion: In real-world clinical deployment, our model expedites reporting of pneumonia in COVID-19 suspects while preserving diagnostic performance without significant model drift.

## 1. Introduction

An outbreak caused by the SARS-CoV-2 (severe acute respiratory syndrome coronavirus 2) was first identified in Wuhan, China in December 2019 [1]. The World Health Organisation first established COVID-19 as a global pandemic on 11 March 2020 [2]. Singapore is an independent city-state with one of the highest testing [3] and vaccination rates in the world [4], with the National Centre for Infectious Diseases (NCID) at the heart of the nation’s response to COVID-19. At the time of writing, Singapore has seen more than 280,000 confirmed cases with 835 COVID-19-related deaths [5].

Although reverse transcription polymerase chain reaction (RT-PCR) is the gold standard for diagnosing COVID-19, the process can be time consuming and sometimes results in delay in diagnosis. Computed tomography (CT) findings have been shown to correlate well with RT-PCR result [6], and since the pandemic began, deep learning algorithms have been developed to diagnose COVID-19 through CT scans [7,8,9] or through predictive models built around laboratory results [10]. However, the American College of Radiology (ACR) cautioned against using CT as a first-line test to diagnose COVID-19 given the non-specific imaging findings of the disease and the fact that a normal chest CT does not preclude COVID-19 [11]. Thus, most ambulatory care facilities still rely on the chest radiograph as the mainstay of initial radiological investigation in COVID-19 screening workflows, as these are more easily acquired, and X-ray machines are logistically more amenable to infection control measures.

The clinical value of CXRs is less for diagnosis of COVID-19 but more for identification of imaging signs of pneumonia and assessment of severity for clinical decision making, as more groups utilise CXRs to analyse the severity of disease and to predict clinical outcome [12,13,14]. Published recommendations of the Flesichner Society [15] and the European Society for Thoracic Imaging are in line with this approach [16]. Several groups have applied deep learning methods for detection of COVID-19 pneumonia on CXRs often with accuracies of over 90% [17,18,19,20,21,22,23]. However, most of these models were tested in simulated environments, and their diagnostic performance in the real-world setting has not been validated. It is known that the diagnostic performance of algorithms can degrade significantly when deployed in clinical practice [24]. Furthermore, the clinical impact of these image-based deep learning models is not often measured in the same setting.

At the height of the first wave of the pandemic in 2020, our team developed a deep learning model trained with CXRs acquired in a specialised COVID-19 national screening facility and assessed its ability to detect pneumonia in COVID-19 suspects. To take it a few steps further, we assessed and validated the model’s diagnostic performance in a real-world setting before deploying and integrating the model into our daily clinical workflow. In this study, we measured its performance on a “live” dataset and further evaluated its impact on the turn-around time.

## 2. Materials and Methods

This study was approved by the institutional review boards of the respective institutions and compliant with the Health Insurance Portability and Accountability Act (HIPAA). A waiver of consent was granted due to the retrospective nature of the study and minimal risks involved.

### 2.1. Datasets

There were three separate datasets included in this study, including the training set, proof-of-concept (POC) offsite test set, and the “live” clinical deployment set. All CXRs were acquired in the same hospital and re-sized to a resolution of 224 × 224 pixels. Two senior radiologists (each with more than a decade of experience) provided the reference standard. Any disagreement was resolved through discussion of perceived radiological findings and through consultation with two other senior radiologists not directly involved in the study. The team chose to adopt radiologists’ inputs as reference standard (instead of PCR results), as the goal of this project was to create a tool to carry out high-throughput screening to triage COVID-19 suspects with radiologically proven pneumonia; as such, approximating the model’s performance to that of senior radiologists was deemed more relevant. Furthermore, we know that most patients with COVID-19 do not develop pneumonia, especially earlier on in the disease [25]. Therefore, as a point to note, in the following datasets, the “pneumonia-negative” groups may contain patients who have tested positive on the SARS-CoV-2 RT-PCR test given that the ground-truth labels are radiological observations. The converse is also true.

#### 2.1.1. Training Set

The training dataset comprised of frontal CXRs acquired in NCID and NCID’s partner hospital. A total of 4277 radiographs were included; 971 of these were ground truth-labelled positive for pneumonia. Out of these pneumonia-positive radiographs, 465 were culture-confirmed bacterial pneumonia or non-COVID-related viral pneumoniae, while the other 506 were RT-PCR confirmed COVID-19 cases. Cases that were labelled negative for pneumonia constituted the negative group even if (1) there were other abnormalities noted on the chest radiograph (e.g., pneumothorax, atelectasis, etc.), or (2) they tested positive on the COVID-19 RT-PCR test. One-fifth of the training set was held out as the validation set.

#### 2.1.2. Proof-of-Concept (POC) Offsite Test Set

A total of 1440 frontal CXRs acquired in NCID were used as an offsite test dataset. Seventy-two of these radiographs were ground truth-labelled positive for pneumonia. With regards to demographics, 84% of these patients were male (*n* = 1209), while 16% were female (*n* = 231), with an average age of 35.8.

#### 2.1.3. Clinical Deployment Set

The clinical deployment set included 3714 frontal CXRs acquired in NCID, with 189 ground truth-labelled as positive for pneumonia. In terms of demographics, 87% of these patients were male (*n* = 3241), while 13% were female (*n* = 473). The average age of the patient population for this dataset was 37. Both the offsite test and the clinical deployment datasets were predominantly made up of young, adult males because Singapore was experiencing a surge in cases in the foreign worker dormitories at the time the deployment was carried out.

All patients in the latter two dataset were patients suspected of having COVID-19 at the time the CXR was acquired. A large majority of the CXRs in these two datasets were notably unremarkable, as we exclusively utilised CXRs that were acquired in NCID, which did not attend to patients with non-COVID-related complaints.

While the reference standard we adopted was input from senior radiologists, we collected the SARS-CoV-2 RT-PCR results from all COVID-suspect patients involved in the study. This is presented in Appendix A.

### 2.2. Development of the Deep Learning Model

To rapidly deploy a deep learning model for COVID-19 screening, we constructed and tested an ensemble model with the training dataset. We leveraged on transfer learning based on existing trained networks in view of the relative urgent need for increased efficiency in times of rapidly evolving global pandemic. Several groups have also tapped into transfer learning to create models for detecting COVD-19 on X-rays, CT, and even ultrasound [26,27].

#### 2.2.1. Transfer Learning on Deep Neural Networks

Transfer learning is a technique for predictive modelling on a different but somehow similar problem that can then be reused partly or wholly to accelerate the training and improve the performance of a machine learning model [28]. The most commonly used transfer learning technique for deep neural network strategies are pre-training based methods [29]. We utilised the weight initialisation-based method, a common technique used in medical image analysis tasks, which reuses the trained weights on a source dataset as a start point for the target dataset. In this manner, all the weights should be adjusted for the target task in the fine-tuning step.

#### 2.2.2. Network Architectures

**Figure 1:**
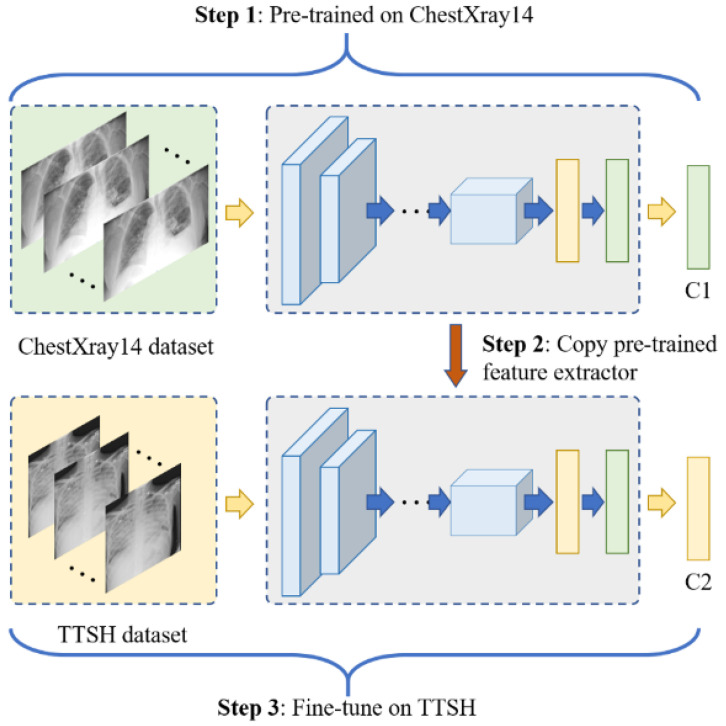
Workflow of pre-training, initializing, and fine-tuning processes. C1 is a multi-label classifier with 14 elements, and C2 is a classifier containing two neurons.

We employed the Dense CNN (DenseNet [30]) as the backbone model to distinguish CXRs. The workflow of pre-training, initializing, and fine-tuning processes is shown in Figure 1. In pre-training (Step 1), the networks are firstly trained on the publicly available ChestX-ray14 dataset [31] to obtain some common features. In Step 2, the pre-trained weights except the last layer are used to initialise the DenseNet121 model for the hospital’s dataset. For the final step, we fine-tuned all the layers in the DenseNet121 model using our hospital’s dataset. To exploit data distribution and representation, our team created several different models for data ensemble using transfer learning, focal loss, weight cross entropy loss, and model adaptation. We expected data imbalance between the two classes (negative or positive for pneumonia), as most of the screening CXRs were likely to be normal. To tackle this issue of data imbalance, we utilised data augmentation through multiple sampling of the data and weighted loss to ensure representative results. The technical details of the network architecture are elaborated upon in Appendix B.

### 2.3. Deployment of Model Ensemble

**Figure 2:**
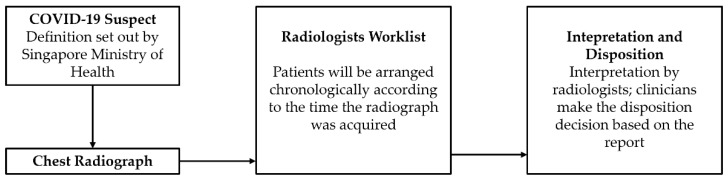
NCID Screening Centre Workflow.

There are several approaches and flowcharts in diagnosing and ruling out COVID-19, and CXRs have been widely used as an integral part of the triage process [15,32]. NCID screening centre utilizes a similar workflow, depicted in Figure 2. The clinicians and radiologists further agreed upon a 1-h turn-around time (TAT) for interpretation of all CXRs done in NCID to facilitate patient flow and to minimize chokepoints [33]. The team calculated the TAT by extracting timestamps from the hospital’s Centricity Radiology Information System (RIS) and computing the time elapsed between the completion of the CXR and final approval of the CXR report.

We deployed the model in an Ubuntu 18.04 virtual machine (VM) hosted in a Windows 10 workstation with the following hardware specifications: Intel Xeon Gold 6242 processor, 128 GB RAM, and 2 X NVIDIA GeForce RTX 2080 TI GPU, located in the hospital’s Department of Diagnostic Radiology. The VM was configured as a Generation 2 Hyper-V VM with 8 virtual processors and a fixed 16 GB RAM.

The hospital uses the Centricity Radiology Information System (RIS) and Picture Archiving and Communication System (PACS) from GE Healthcare. A mini-PACS, RA600 (GE Healthcare, Chicago, IL, USA), was installed in a workstation serving as a DICOM listener to temporarily store DICOM files independently from the hospital’s PACS system.

The X-ray modality used in NCID is the FDR Visionary Suite (Fujifilm Healthcare, Tokyo, Japan). These machines were configured to send the CXRs to the hospital’s PACS and the RA600. Only frontal CXRs (anteroposterior and posteroanterior) were sent to the RA600; other projections were excluded. The RA600 receives and stores incoming DICOM files sent to the workstation. The local DICOM store is exported as a shared folder and mounted in the VM via the Server Message Block (SMB) protocol. From here, the model retrieves and interprets each CXR and assigns a binary value, either “0” for absence of pneumonia or “1” for the presence of pneumonia. The radiograph is then deleted from the workstation after processing to minimise the risk of unauthorised access to patients’ identifiers. With regards to computational cost, the model takes less than 3 s for one image using all the models running on a CPU workstation (Intel Xeon Gold 6242, 2.8 GHz, RAM 256 G). In batch processing using GPU, it takes less than 0.01 s per image per model.

**Figure 3:**
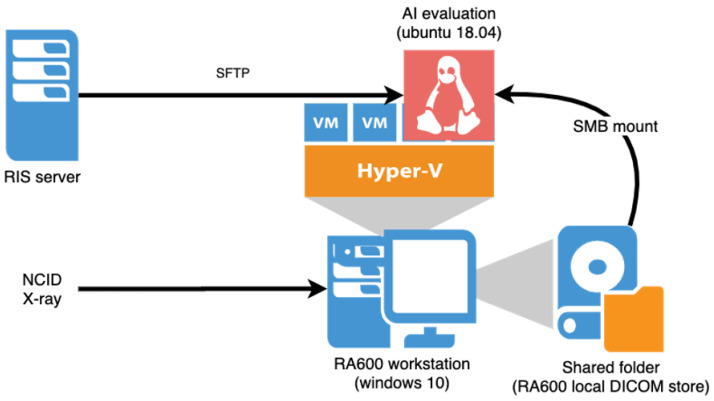
AI Model Deployment Infrastructure.

Finally, the results were retrieved via SSH File Transfer Protocol (SFTP) and updated automatically into the RIS system and matched by each case’s accession number as the unique identifier. Cases that were flagged as positive by the algorithm were prioritized to the top of the radiologist worklist chronologically. This was done using a Health Level Seven (HL7) compliant script developed by the hospital’s RIS/PACS vendor. A diagrammatic illustration of the deployment process is shown in Figure 3. Of note, this method merely provided the prediction of the model to the reporting radiologists; the responsibility of making the final diagnosis still rested with the radiologists.

To ensure our infrastructure complied with the hospital’s security standards, official approval was obtained from the Singapore’s Integrated Health Information Systems (IHiS) committee overseeing the hospital’s IT risk and security.

### 2.4. Proof of Concept (POC)—Offsite Test

Once our team had the model and infrastructure in place, we conducted an offsite test over the course of a week to ensure satisfactory model performance before officially incorporating the model into the daily workflow. The results of this offsite test are presented in a later segment.

### 2.5. Statistical Analysis

As the networks were trained with the probability as output, we used binary cross entropy or focal loss based on class probability to classify a CXR as pneumonia or non-pneumonia. We could then use a threshold on the network output to obtain different sensitivities and specificities to form the ROC curve on any testing dataset. The performance of the algorithm was expressed in AUC, F1 score, sensitivity, specificity, and accuracy. The 95% confidence intervals (CIs) were computed using MATLAB R2014b. The CIs were computed by specific functions within the MATLAB interface (perfcurve and bootci) and used the bias-corrected and accelerated percentile method [34].

## 3. Results

### 3.1. Results from Proof of Concept—Offsite Test Set

**Figure 4:**
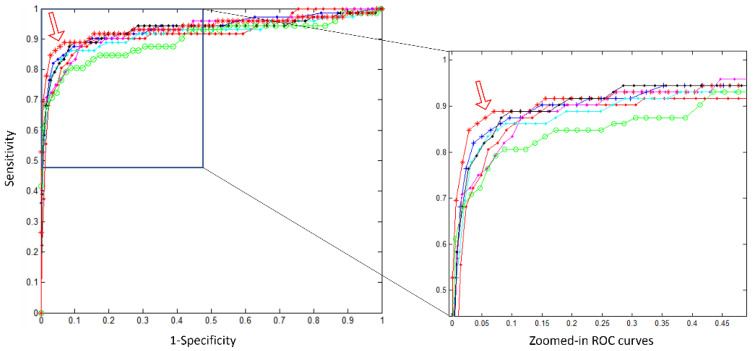
ROC curves for the 7 models and the ensemble model on the POC offsite test. The AUCs of individual models are 0.9185, 0.9355, 0.9265, 0.9163, 0.9285, 0.8976, and 0.9120. The ensemble AUC = 0.9369 (marked by red *, indicated by the arrow in the image). The highest individual model DenseNet121 has an AUC = 0.9355 (shown in blue + line).

**Figure 5:**
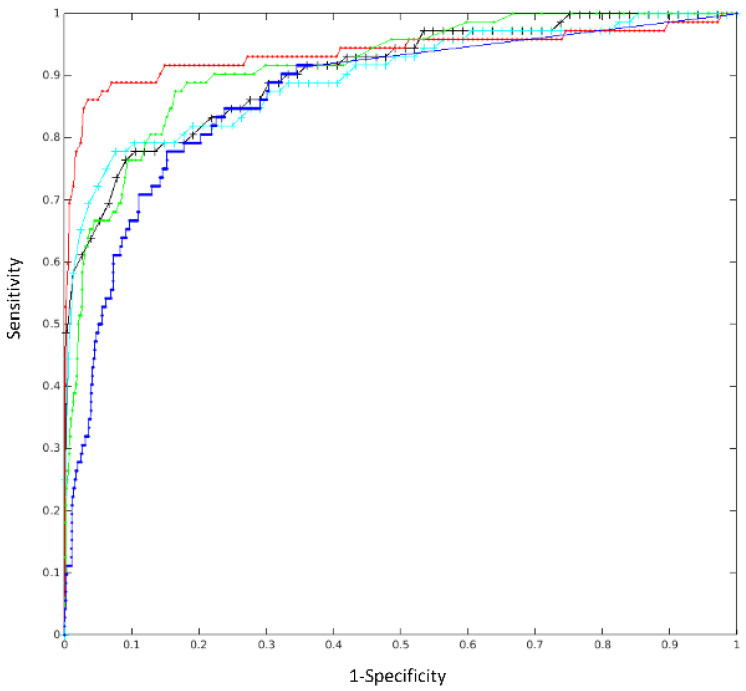
POC test result: ROC curves for the proposed ensemble model (in red, AUC = 0.9369, 95% CI (0.8755, 0.9687)) and other models from the following: Oh et al. (patch-based) [35] in green, AUC = 0.9144; Chen et al. (mmdetection) [36] in blue, AUC = 0.8655; Ozturk et al. (Darknet) [37] in black, AUC = 0.9051; and Minaee et al. (SqueezeNet) [38] in cyan, AUC = 0.9002.

We validated our trained model with different methods and network architectures. Comparing each individual network architecture, the DenseNet121 transfer-learned achieved the best results. Our ensemble of seven models further enhanced the algorithms’ performances; this is presented in Table 1. We compared our ensemble algorithm with existing, published deep learning algorithms using an offsite test dataset as a proof-of-concept (POC). This POC dataset included a total of 1440 CXRs, of which 72 were labelled positive for pneumonia. Our ensemble algorithm achieved the highest AUC (AUC = 0.9369) with maximum F1 of 0.9120. The results of this POC test are depicted in Table 2, with the ROC curves displayed in Figure 4. Incidentally, the patch-based method [35] shows better result when the specificity is low and sensitivity is high, and this is better shown in Figure 5.

### 3.2. Results from Clinical Deployment

**Figure 6:**
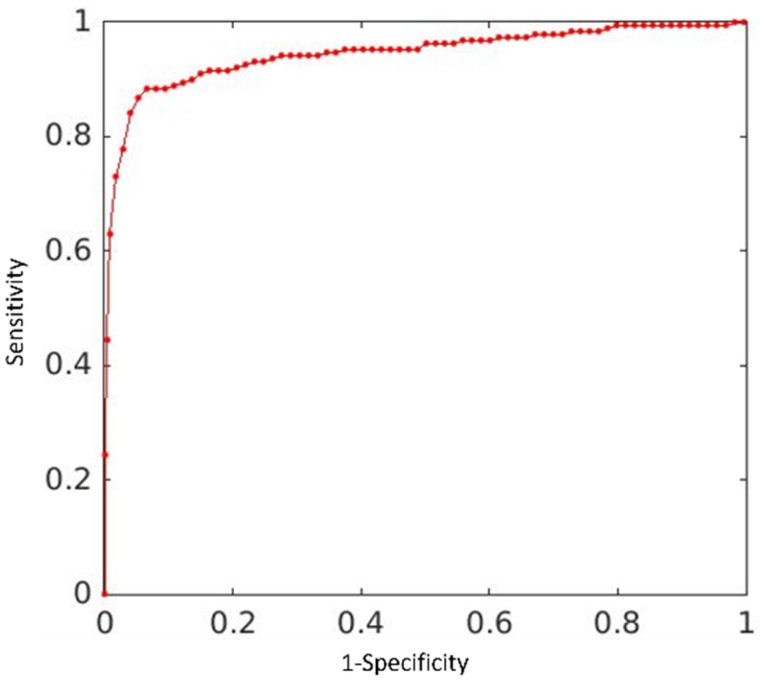
Deployment test result: ROC curves for the proposed ensemble model: AUC = 0.9456, maximum of F1 = 0.9118.

A total of 3714 unique CXRs were included in deployment set, with 189 studies labelled as positive for pneumonia. On this “live” dataset, our algorithm achieved an AUC of 0.9456, 95% CI (0.9181, 0.9627) and maximum F1 of 0.9118. The ROC curve is illustrated in Figure 6. By setting a threshold, we have a confusion matrix, as shown in Table 3.

At the height of the pandemic in Singapore, our team saw a dire need to optimise hospital resources. As such, we opted for a model that did not “over-diagnose” pneumonia, as that may cloud the opinion of the reporting radiologists and result in unnecessary use of scarce healthcare resources. Therefore, we set a threshold that resulted in a higher negative predictive value (NPV).

Our algorithm attained a high specificity of 97.1% (95% CI (0.9664, 0.9760)) while maintaining sensitivity of 78.8% (95% CI (0.7246, 0.8435)) with NPV of 98.8% (95% CI (0.9843, 0.9916)) and positive predictive value (PPV) of 59.1% (95% CI (0.5311, 0.6518)). Appendix C describes three different examples using saliency maps and probability outputs. Appendix A shows the distribution of the RT-PCR test results of this “live” dataset.

### 3.3. Turnaround Time

Turn-around time (TAT) was calculated using the data extracted from the hospital’s RIS. We measured the average TAT within a three-week window before and after the deployment of our deep learning model. Radiologists took an average of seven minutes to complete a report as opposed to nine minutes prior to the deployment, demonstrating a 22% reduction in TAT.

## 4. Discussion

There have been growing efforts from researchers to develop an efficient and reliable AI solution to help diagnose patients with COVID-19. Most published studies related to our subject of interest attained good results but did not go on to test their models in a real-world clinical setting [17,18,19,20,21,22,23,35]. Al-Waisy et al. fused results obtained from two different deep learning methods to achieve accuracy rates of 99.93% [18]. Nayak et al. conducted a comprehensive review of eight pre-trained CNN models and found ResNet-34 to be the best performing model at 98.33% [19]. This study’s model boasted an AUC of 0.95, with specificity of 97.1% and moderate sensitivity of 78.8%. The diagnostic performance was not degraded when deployed in the clinical setting, something which the prior studies have not demonstrated.

As a proof-of value, our group wanted to establish a model that could carry out high-throughput screening for COVID-19 pneumonia in large numbers of suspected COVID-19 patients to quickly triage patients with CXR radiographic findings prior to a RT-PCR diagnosis to stratify for high-risk patient management and optimize hospital resources. This is especially relevant now with the emergence of Delta and now the Omicron variants, as healthcare institutions around the world struggle to allocate scarce healthcare resources [39,40]. This study evaluated the diagnostic performance of our model on a “live” dataset and demonstrated that it is possible to preserve diagnostic performance of a deep learning algorithm when transferred to clinical deployment. This is likely due to an adequate dataset, unbiased in selection and curation, that was used for fine-tuning a base algorithm.

In this study, the deep learning model performed well utilising radiologists’ input as reference standard. We chose to adopt senior radiologists’ inputs as reference standard, as the team envisioned a tool that could help prioritise patients with definite radiological findings and thus alert the clinicians to these patients who are potentially more vulnerable to clinical deterioration [41] and should hopefully receive earlier intervention. Therefore, approximating the model’s performance to that of senior radiologists was deemed more relevant. While the specific features of COVID-19 on CT have been described extensively [42,43], the features of COVID-19 on CXRs are more ambiguous. That said, a frontal CXR remains the mainstay of initial radiological screening in most institutions in this pandemic, making our model relevant for widespread adoption. In our clinical deployment dataset, 607 patients had a positive RT-PCR result, and 48 of these patients had a CXR that was flagged as abnormal by the deep learning algorithm. We postulate the model fared worse than those that have been published [18,19,44,45] because it was deployed in Singapore’s national screening facility where a large majority of the patients were asymptomatic or oligosymptomatic on presentation, with more subtle radiological findings, thus posing a greater challenge to the model. This is largely secondary to the active tracing and aggressive testing policy that Singapore pursues.

In our institution, clinicians and radiologists have agreed upon a 1-h turn-around time (TAT) from the acquisition of a CXR image to the completion of a radiological report. Even though there was a modest 22% improvement in TAT during the deployment period, we believe that the value of the model in increasing radiologist efficiency has not been fully justified given the relatively low number of positive cases at our institution during the clinical deployment phase. As a triaging tool, we believe that the model can provide greater reduction in TAT in healthcare facilities that face large surges in demand for radiologist reporting.

**Figure A2:**
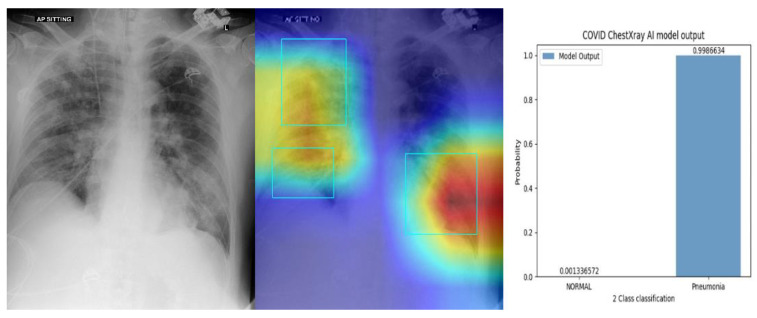
Selected true-positive example. The algorithm correctly diagnosed pneumonia in this case, where bilateral, multifocal airspace opacities are seen.

**Figure A3:**
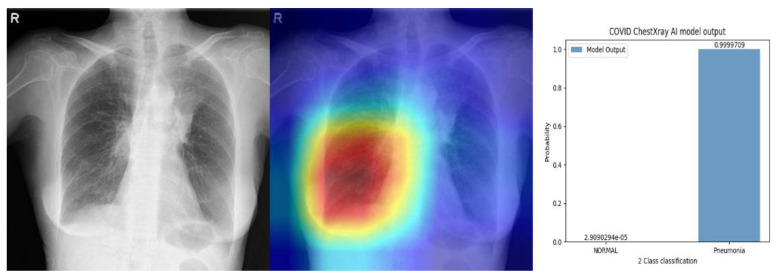
Selected false-positive example. This example demonstrates a potential pitfall in CXRs with prominent breast shadows.

**Figure A4:**
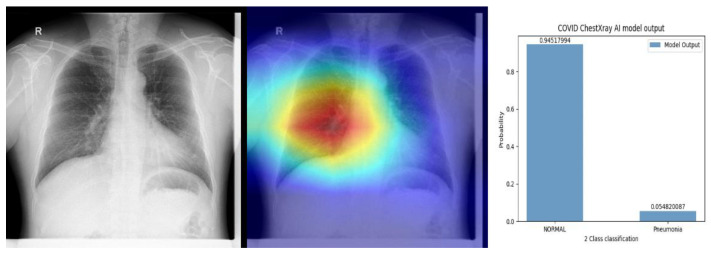
Selected false-negative example. The airspace changes in this case are relatively mild, posing a greater challenge to the algorithm.

The exact impact of the deep learning model on the eventual accuracy of the radiologists is beyond the scope of this study although work is underway in our institution to investigate this aspect further. Several studies have shown that their AI system can identify characteristics of COVID-19 on chest radiographs with performance comparable to experienced radiologists [44,46,47]. Harrison et al. further proved that a well-built AI system improved radiologists’ performance in distinguishing COVID-19 pneumonia from non-COVID-19 pneumonia at chest CT [48]. In addition, the team attempted to employ saliency maps to detect features characteristic of COVID-19 on chest radiographs. The saliency maps indicate the regions of each radiograph that had the greatest influence on the model’s prediction. We realised that while some of these maps accurately highlight diseased lung fields (See Figure A2 in Appendix C), their presence can be confounding in false-positive, false-negative, or even true-negative cases (See Figure A3 and Figure A4 in Appendix C). Localisation of the pathology is not the main desired output of our network, but the utility of saliency maps in localisation models (e.g., detecting lung nodules on CT) warrants additional scrutiny.

Zandehshahvar et al. reported a deep learning approach to analyse severity of COVID-19 [12] while Dayan et al. included CXRs as a key component of their clinical outcome predictive model [13]. In future advancements, a radiographic severity score can potentially be integrated into our algorithm with relevant outputs that can alert the clinicians to patients who are more vulnerable to clinical deterioration and therefore intervene earlier.

Even though this was a prospective clinical deployment, there remain several limitations to our study. Firstly, the development, validation, deployment, and testing of the algorithm were all done within a single institution. While working within a single institution hastened the relevant processes, cross-institution deployment would be needed to ensure replicability of our results and reliability of our model. However, the degree of variation is likely to be low given that our COVID-19 screening centre is the largest national facility, and it receives referrals from across our country. Secondly, the most frequently observed distribution patterns of COVID-19 include bilateral involvement, peripheral distribution, consolidations, and ground-glass opacification, whereas pleural effusions are rare [49,50]. Many of the features are also seen in bacterial and other viral pneumoniae; hence, it is not certain if our model can reliably differentiate patients with community acquired pneumonias from those of COVID-19. Thirdly, our algorithm is purely based on computer vision even though we know that in clinical practice, history of exposure, patient’s symptoms, and laboratory results are important factors considered as part of the clinical diagnostic workup. Finally, our model was trained, validated, and tested on adult CXRs. As such, these results cannot be extrapolated to the paediatric population. Fortunately, only a small minority of the known COVID-19 cases are in children, and most of the confirmed cases in the paediatric population have relatively milder symptoms [51] and likely minimal CXR findings.

## 5. Conclusions

In conclusion, even as the global pandemic of COVID-19 evolves, chest radiography remains a valuable tool in the screening and severity assessment of disease. A reliable and readily deployable AI algorithm can expedite clinical decision making. Our team developed a deep learning algorithm that performs well in a simulated environment and preserves its diagnostic performance when tested with a “live” dataset in real-world clinical deployment. The algorithm was integrated into the clinical workflow and successfully reduced report turn-around time by prioritising abnormal cases.

Future work can incorporate non-image-related data into a single model to further improve performance and could involve cross-institution deployment to ensure replicability and reliability. Our team also aims to measure improvement in radiologists’ performance (if any) following augmentation with our deep learning model.

## Figures and Tables

**Table 1:** Offsite Test with 1440 CXRs: results of individual models versus model ensemble.

Model	1	2	3	4	5	6	7	Ensemble
AUC	0.9185	0.9120	0.9355	0.9265	0.9163	0.9286	0.8976	0.9369
F1	0.8835	0.8587	0.8938	0.8867	0.8906	0.8981	0.8558	0.9120

**Table 2:** Proof of Concept: Results from Offsite Test compared with other published models.

	ROC AUC	Max F1 Score
Oh et al. (Patch-based) [35]	0.9144	0.8544
Chen et al. (MMDetection) [36]	0.8685	0.8110
Ozturk et al. (Darknet) [37]	0.9051	0.8344
Minaee et al. (SqueezeNet) [38]	0.9002	0.8464
Best performing single DenseNet121 network	0.9355	0.8981
Ensemble of seven models	0.9369	0.9120

**Table 3:** Confusion matrix on the Clinical Deployment Dataset.

AI Prediction	Positive	Negative
Ground Truth		
**CXR Positive**	149	40
**CXR Negative**	103	3422

**Figure A1:**
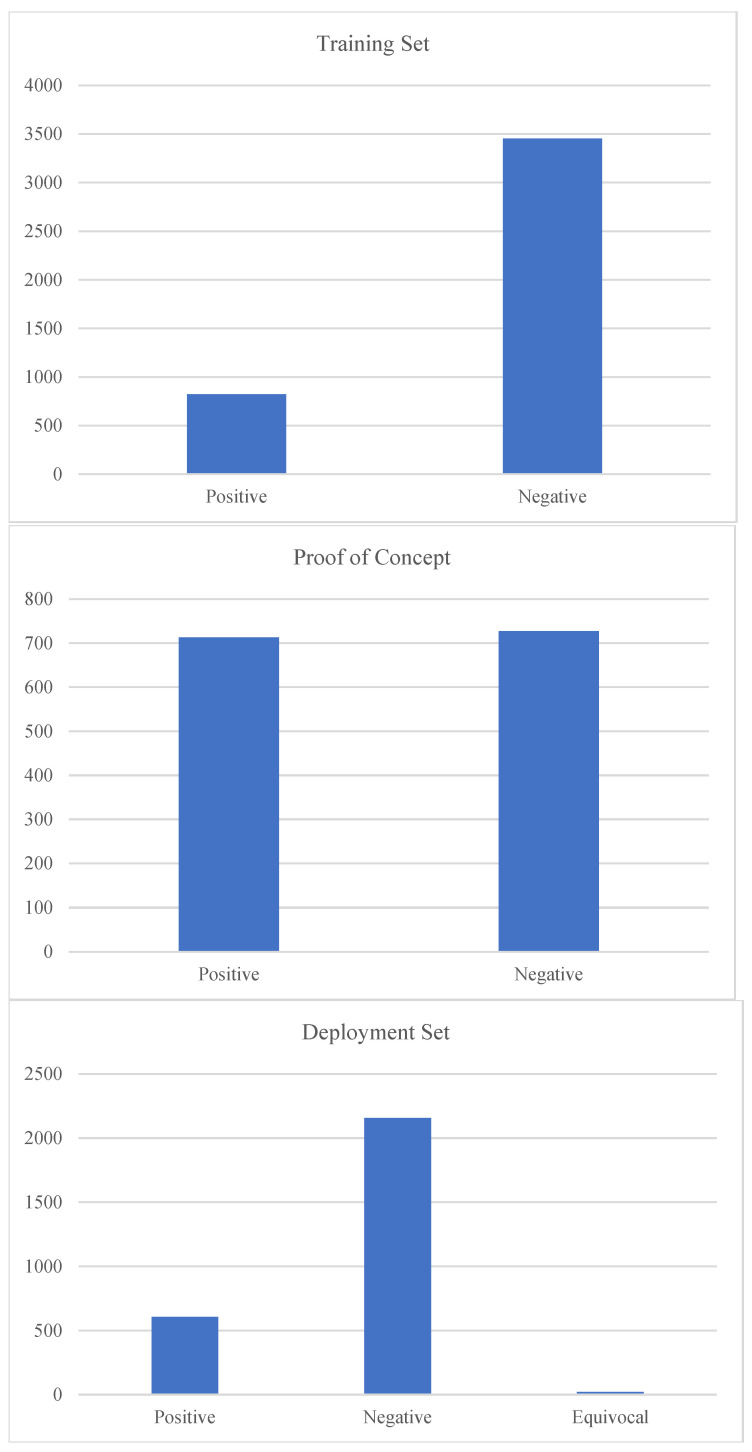
Distribution of SARS-CoV-2 RT-PCR Test Results of training set, Proof-of-Concept and Deployment datasets.

## Data Availability

Not applicable.

## Appendix B. Technical Details of the Network Architecture and Performance Matrix

In brief, DenseNets are densely connected neural networks comprised of Dense Blocks and transition layers with convolutional layers and a max-pooling that connect each sequential block. In each Dense Block, there can be varying groups of [1 × 1] and [3 × 3] paired convolutional layers, with a pre-processing layer of Batch Normalization (BN) and rectified linear unit (ReLU). The original design of the DenseNet121 has a global averaging pooling layer followed by a 1000-node fully connected (FC) layer for feature mapping.

### Pre-Training and Fine-Tuning

In the pre-training phase (Step 1), the model is first trained on a large scale dataset (ChestXray14), which encompassed 14 common thoracic diseases, including pneumonia, cardiomegaly, pneumothorax, etc. [31]. We utilised a 4:1 training/validation split on the NIH dataset. We optimised the Binary Cross Entropy (BCE) with Logits Loss, which combines a sigmoid layer and the BCE Loss in each class.

In the fine-tuning phase (Step 3), we optimised the Weighted Cross Entropy Loss (WCEL) for the classifier (C2) on the target dataset. For a single sample $x_{i}$ in our TTSH dataset, we used $y_{i}$ to denote the corresponding label, and $y_{i}={\left[0,1\right]}^{T},\;$ or $y_{i}={\left[1,0\right]}^{T}$. The WCEL can thus be defined as:

(A1) $$L\;\left(x_{i},y_{i}\right)=-\left(y_{1i}\log\left({\hat{y}}_{1i}\right)+uy_{2i}\log\left({\hat{y}}_{2i}\right)\right),$$

where *u* is a manual rescaling weight given to the positive class; we set *u* to be the ratio of the number of negative training samples to the number of positive training samples in this work. ${\hat{y}}_{i}$ denotes the network’s prediction for the *i*th input image $x_{i}$ from the target domain.

The network is trained end-to-end using Adam [52] with standard parameters (0.9, 0.999). The batch size of our model is 32. We used an initial learning rate of 0.0001 and picked the model with the highest Area Under the Receiver Operating Characteristic (ROC) Curve (AUC) score on the validation set.

To diversify the model representative, we adjusted the CheXnet14 network (Dense121—121 layers of networks) with two fully connected (FC) layers (added two FCs, with 1024 nodes and 128 nodes, respectively, following a sigmoid node for classification) in the classification end (C2 in Figure 1) and experimented with different loss function at network tuning stage. In previous renditions of DenseNet121, Wang et al. adopted a weighted cross entropy loss [31], while Rajpurkar et al. utilised an unweighted binary cross entropy loss for network training. [53] To enhance the capability, we modified the loss function for the DenseNet121 with a Focal Loss (FL), [54] which is designed to handle the large data imbalance between classes. We also carried our data augmentation through multiple sampling to better represent the random distribution from the available dataset. We added an experiment to compare the results before and after data augmentation and weighted loss and found that the latter effectively improved the classification results. This is especially relevant in national screening centres, where most of the screening CXRs are expected to be normal. We also increased the FC layers after feature extraction to give us better distinguishing power for the classification of CXRs with features suggestive of COVID-19. The FL is defined by

*FL*(*p_t_*) = −*α*(1 − *p_t_*) *^γ^* log(*p_t_*) (A2)

where *p_t_ =* $\{\begin{array}{c} p,\;if\;y=1\\ 1-p,\;if\;y=0 \end{array}$, *y* is the ground truth of the class, and *p* is the estimation output of the network for an input CXR image. We adopted Pydicom [55] for image pre-processing. We set each CXR image mean to 0 and standard deviation to 1 for image intensity normalization. The parameter for Focal Loss is set as *α* = 0.25, *γ* = 2. Thus far, we utilised model adaptation and WCEL (Equation (A1), Model A) and transfer learning and FL (Equation (A2), Model B) to refine two models using the TTSH training dataset. To exploit the data distribution and representation, we built another five models for data ensemble, using Model B as backbone model, as it has better performance in AUC and F1. The five models were trained using five sub datasets randomly sampled from the TTSH training data. Experiments showed that with the model ensemble and data ensemble, the overall AUC and F1 outperformed any single model (Model A and Model B) or combination of them.

Following with the terms TP, TN, FP, and FN, we can further define the performance matrix.

- TP: true prediction on positive cases,
- TN: true prediction on negative cases,
- FN: false prediction on positive cases,
- FP: false prediction on negative cases,
- Sensitivity = TP/(TP + FN),
- Specificity = TN/(TN + FP),
- F1 is the harmonic mean of Sensitivity and Specificity,
- F1 = 2 × Sensitivity × Specificity/(Sensitivity + Specificity), and
- Accuracy = (TP + TN)/(TP + FN + TN + FP).

## Appendix C. Examples Using Saliency Maps and Probability Output

The first image of each example shows the annotation by our radiologists, the second image demonstrates the saliency map generated for that image by our algorithm, and the last image shows the probability score of the algorithm’s diagnosis. Of note, the red areas in the saliency maps display areas of interest and may not necessarily focus on the area of abnormality.

## References

[1] Chen N., Zhou M., Dong X., Qu J., Gong F., Han Y., Qiu Y., Wang J., Liu Y., Wei Y. (2020). Epidemiological and clinical characteristics of 99 cases of 2019 novel coronavirus pneumonia in Wuhan, China: A descriptive study. Lancet.

[2] WHO (2020). WHO Director-General’s Opening Remarks at the Media Briefing on COVID-19—11 March 2020. https://www.who.int/dg/speeches/detail/who-director-general-s-opening-remarks-at-the-media-briefing-on-covid-19---11-march-2020.

[3] Goh T. (2020). Coronavirus: Singapore’s Testing Rate Is Tops in Asean, with Over 1 m Swabs Done, the Straits Times. https://www.straitstimes.com/singapore/health/spores-testing-rate-is-tops-in-asean-with-over-1m-swabs-done.

[4] Tracking Singapore’s COVID-19 Vaccination Progress | The Straits Times, the Straits Times. https://www.straitstimes.com/multimedia/graphics/2021/06/singapore-covid-vaccination-tracker/index.html?shell.

[5] Ministry of Health Updates on COVID-19 (Coronavirus Disease 2019) Local Situation. https://www.moh.gov.sg/covid-19.

[6] Ai T., Yang Z., Hou H., Zhan C., Chen C., Lv W., Tao Q., Sun Z., Xia L. (2020). Correlation of Chest CT and RT-PCR Testing in Coronavirus Disease 2019 (COVID-19) in China: A Report of 1014 Cases. Radiology.

[7] Wang M., Xia C., Huang L., Xu S., Qin C., Liu J., Cao Y., Yu P., Zhu T., Zhu H. (2020). Deep learning-based triage and analysis of lesion burden for COVID-19: A retrospective study with external validation. Lancet Digit Health.

[8] Jaiswal A., Gianchandani N., Singh D., Kumar V., Kaur M. (2020). Classification of the COVID-19 infected patients using DenseNet201 based deep transfer learning. J. Biomol. Struct. Dyn..

[9] Voulodimos A., Protopapadakis E., Katsamenis I., Doulamis A., Doulamis N. Deep learning models for COVID-19 infected area segmentation in CT images. Proceedings of the 14th PErvasive Technologies Related to Assistive Environments Conference.

[10] Alakus T.B., Turkoglu I. (2020). Comparison of deep learning approaches to predict COVID-19 infection. Chaos Solitons Fractals.

[11] American College of Radiology (2020). ACR Recommendations for the Use of Chest Radiography and Computed Tomography (CT) for Suspected COVID-19 Infection. https://www.acr.org/Advocacy-and-Economics/ACR-Position-Statements/Recommendations-for-Chest-Radiography-and-CT-for-Suspected-COVID19-Infection.

[12] Zandehshahvar M., van Assen M., Maleki H., Kiarashi Y., de Cecco C.N., Adibi A. (2021). Toward understanding COVID-19 pneumonia: A deep-learning-based approach for severity analysis and monitoring the disease. Sci. Rep..

[13] Dayan I., Roth H.R., Zhong A., Harouni A., Gentili A., Abidin A.Z., Liu A., Costa A.B., Wood B.J., Tsai C.-S. (2021). Federated learning for predicting clinical outcomes in patients with COVID-19. Nat. Med..

[14] Wynants L., van Calster B., Collins G.S., Riley R.D., Heinze G., Schuit E., Bonten M.M.J., Dahly D.L., Damen J.A., Debray T.P.A. (2020). Prediction models for diagnosis and prognosis of COVID-19: Systematic review and critical appraisal. BMJ.

[15] Rubin G., Ryerson C., Haramati L. (2020). The Role of Chest Imaging in Patient Management during the COVID-19 Pandemic: A Multinational Consensus Statement from the Fleischner Society. Radiology.

[16] Revel M.P., Parkar A.P., Prosch H., Silva M., Sverzellati N., Gleeson F., Brady A. (2020). COVID-19 patients and the radiology department—Advice from the European Society of Radiology (ESR) and the European Society of Thoracic Imaging (ESTI). Eur. Radiol..

[17] Hammoudi K., Benhabiles H., Melkemi M., Dornaika F., Arganda-Carreras I., Collard D., Scherpereel A. (2020). Deep Learning on Chest X-ray Images to Detect and Evaluate Pneumonia Cases at the Era of COVID-19. J. Med. Syst..

[18] Al-Waisy A.S., Mohammed M.A., Al-Fahdawi S., Maashi M.S., Garcia-Zapirain B., Abdulkareem K.H., Mostafa S.A., Kumar N.M., Le D.N. (2021). COVID-DeepNet: Hybrid Multimodal Deep Learning System for Improving COVID-19 Pneumonia Detection in Chest X-ray Images. Comput. Mater. Contin..

[19] Nayak S.R., Nayak D.R., Sinha U., Arora V., Pachori R.B. (2021). Application of deep learning techniques for detection of COVID-19 cases using chest X-ray images: A comprehensive study. Biomed. Signal Process. Control..

[20] Zhang J., Xie Y., Li Y., Shen C., Xia Y. (2020). COVID-19 Screening on Chest X-ray Images Using Deep Learning based Anomaly Detection. arXiv.

[21] Borkowski A., Viswanadhan N., Thomas B., Guzman R.D., Deland L.A., Mastorides S.M. (2020). Using Artificial Intelligence for COVID-19 Chest X-ray Diagnosis. Fed. Pract..

[22] Basu S., Mitra S., Saha N. Deep Learning for Screening COVID-19 using Chest X-ray Images. Proceedings of the 2020 IEEE Symposium Series on Computational Intelligence (SSCI).

[23] Yoo S.H., Geng H., Chiu T.L., Yu S.K., Cho D.C., Heo J., Choi M.S., Choi I.H., Van C.C., Nhung N.V. (2020). Deep Learning-Based Decision-Tree Classifier for COVID-19 Diagnosis from Chest X-ray Imaging. Front. Med..

[24] Kitamura G., Deible C. (2020). Retraining an open-source pneumothorax detecting machine learning algorithm for improved performance to medical images. Clin. Imaging..

[25] Cleverley J., Piper J., Jones M.M. (2020). The role of chest radiography in confirming COVID-19 pneumonia. BMJ.

[26] Horry M.J., Chakraborty S., Paul M., Ulhaq A., Pradhan B., Saha M., Shukla N. (2020). COVID-19 Detection through Transfer Learning Using Multimodal Imaging Data. IEEE Access.

[27] Maghdid H.S., Asaad A.T., Ghafoor K.Z., Sadiq A.S., Khan M.K. (2020). Diagnosing COVID-19 Pneumonia from X-ray and CT Images using Deep Learning and Transfer Learning Algorithms. arXiv.

[28] Thrun S., Pratt L. (2012). Learning to Learn.

[29] Torrey L., Shavlik J. (2010). Handbook of Research on Machine Learning Applications and Trends: Algorithms, Methods, and Techniques.

[30] Huang G., Weinberger K.Q. (2016). Densely Connected Convolutional Networks. arXiv.

[31] Wang X., Peng Y., Lu L., Lu Z., Bagheri M., Summers R.M. ChestX-ray8: Hospital-scale Chest X-ray Database and Benchmarks on Weakly-Supervised Classification and Localization of Common Thorax Diseases. Proc. IEEE Conf. Comput. Vis. Pattern Recognit..

[32] Yang W., Sirajuddin A., Zhang X., Liu G., Teng Z., Zhao S. (2020). The role of imaging in 2019 novel coronavirus pneumonia (COVID-19). Eur. Radiol..

[33] Manauis C.M., Loh M., Kwan J., Mingzhou J.C., Teo H.J., Peng D.T.K., Sushilan S.V., Leo Y.S., Hou A. (2020). Bracing for impact: Operational upshots from the National Centre for Infectious Diseases Screening Centre (Singapore) during the COVID-19 outbreak. J. Am. Coll. Emerg. Phys. Open..

[34] DiCicio T.J., Efron B. (1996). Bootstrap confidence intervals. Stat. Sci..

[35] Oh Y., Park S., Ye J.C. (2020). Deep Learning COVID-19 Features on CXR using Limited Training Data Sets. IEEE Trans. Med. Imaging.

[36] Chen K., Wang J., Pang J., Cao Y., Xiong Y., Li X., Zhu C., Cheng T., Zhao Q., Li B. (2019). MMDetection: Open MMLab Detection Toolbox and Benchmark. arXiv.

[37] Ozturk T., Talo M., Yildirim E.A., Baloglu U.B., Yildirim O., Acharya U.R. (2020). Automated detection of COVID-19 cases using deep neural networks with X-ray images. Comput. Biol. Med..

[38] Minaee S., Kafieh R., Sonka M., Yazdani S., Soufi G.J. (2020). Deep-COVID: Predicting COVID-19 from chest X-ray images using deep transfer learning. Med. Image Anal..

[39] Torjesen I. (2021). COVID-19: Delta variant is now UK’s most dominant strain and spreading through schools. BMJ.

[40] Bolze A., Cirulli E.T., Luo S., White S., Cassens T., Jacobs S., Nguyen J., Iii J.M.R., Sandoval E., Wang X. (2021). Rapid displacement of SARS-CoV-2 variant B.1.1.7 by B.1.617.2 and P.1 in the United States. MedRxiv.

[41] Yasin R., Gouda W. (2020). Chest X-ray findings monitoring COVID-19 disease course and severity. Egypt. J. Radiol. Nucl. Med..

[42] Bernheim A., Mei X., Huang M., Yang Y., Fayad Z.A., Zhang N., Diao K., Lin B., Xiqi Z., Li K. (2020). Chest CT findings in Coronavirus Disease 2019 (COVID-19): Relationship to Duration of Infection. Radiology.

[43] Prokop M., van Everdingen W., Vellinga T.V., van Ufford H.Q., Stöger L., Beenen L., Geurts B., Gietema H., Krdzalic J., Schaefer-Prokop C. (2020). CO-RADS: A Categorical CT Assessment Scheme for Patients Suspected of Having COVID-19-Definition and Evaluation. Radiology.

[44] Murphy K., Smits H., Knoops A.J., Korst M.B., Samson T., Scholten E.T., Schalekamp S., Schaefer-Prokop C.M., Philipsen R.H., Meijers A. (2020). COVID-19 on the Chest Radiograph: A Multi-Reader Evaluation of an AI System. Radiology.

[45] Brunese L., Mercaldo F., Reginelli A., Santone A. (2020). Explainable Deep Learning for Pulmonary Disease and Coronavirus COVID-19 Detection from X-rays. Comput. Methods Programs Biomed..

[46] Zhang R., Tie X., Qi Z., Bevins N.B., Zhang C., Griner D., Song T.K., Nadig J.D., Schiebler M.L., Garrett J.W. (2020). Diagnosis of COVID-19 Pneumonia Using Chest Radiography: Value of Artificial Intelligence. Radiology.

[47] Wehbe R.M., Sheng J., Dutta S., Chai S., Dravid A., Barutcu S., Wu Y., Cantrell D.R., Xiao N., Allen B.D. (2021). DeepCOVID-XR: An artificial intelligence algorithm to detect COVID-19 on chest radiographs trained and tested on a large U.S. Clinical data set. Radiology.

[48] Bai H.X., Wang R., Xiong Z., Hsieh B., Chang K., Halsey K., Tran T.M.L., Choi J.W., Wang D.-C., Shi L.-B. (2020). AI Augmentation of Radiologist Performance in Distinguishing COVID-19 from Pneumonia of Other Etiology on Chest CT. Radiology.

[49] Wong H.Y.F., Lam H.Y.S., Ho-Tung Fong A., Leung S.T., Chin T.W., Lo C.S.Y., Lui M.M., Lee J.C.Y., Chiu K.W., Chung T. (2020). Frequency and Distribution of Chest Radiographic Findings in COVID-19 Positive Patients. Radiology.

[50] Salehi S., Abedi A., Balakrishnan S., Gholamrezanezhad A. (2020). Coronavirus disease 2019 (COVID-19): A systematic review of imaging findings in 919 patients. Am. J. Roentgenol..

[51] Lu X., Zhang L., Du H., Zhang J., Li Y.Y., Qu J. (2020). SARS-CoV-2 Infection in Children. N. Engl. J. Med..

[52] Kingma D.P., Ba J. (2014). Adam: A method for stochastic optimization. arXiv.

[53] Rajpurkar P., Irvin J., Zhu K., Yang B., Mehta H., Duan T., Ding D., Bagul A., Ball R.L., Langlotz C. (2017). CheXNet: Radiologist-Level Pneumonia Detection on Chest X-Rays with Deep Learning. arXiv.

[54] Lin T., Goyal P., Girshick R., He K., Dollar P. (2020). Focal Loss for Dense Object Detection. IEEE Trans. Pattern Anal. Mach. Intell..

[55] Mason D.L. (2008). Pydicom: An Open Source DICOM Library. https://github.com/pydicom/pydicom.

